# A Versatile Chemo-Enzymatic Conjugation Approach Yields Homogeneous and Highly Potent Antibody-Drug Conjugates

**DOI:** 10.3390/ijms18112284

**Published:** 2017-10-31

**Authors:** Ying Xu, Shijie Jin, Wenbin Zhao, Wenhui Liu, Ding Ding, Jie Zhou, Shuqing Chen

**Affiliations:** 1 Zhejiang Provincial Key Laboratory of Anti-Cancer Drug Research, Institute of Drug Metabolism and Drug Analysis, College of Pharmaceutical Sciences, Zhejiang University, Hangzhou 310058, China; 21519008@zju.edu.cn (Y.X.); pharmacy_zwb@zju.edu.cn (W.Z.); liuwenhui@zju.edu.cn (W.L.); 2 College of Pharmaceutical Sciences, Zhejiang Chinese Medical University, Hangzhou 310053, China; shijiejin1997@gmail.com; 3 Formulation and Analysis Laboratory, HisunPharma (Hangzhou) Co. Ltd., Xialian Village, Xukou Town, Fuyang, Hangzhou 311404, China; dingding@hisunpharm.com

**Keywords:** antibody-drug conjugate, site-specific, chemo-enzymatic conjugation, click chemistry

## Abstract

The therapeutic efficacy of antibodies can be successfully improved through targeted delivery of potent cytotoxic drugs in the form of antibody-drug conjugates. However, conventional conjugation strategies lead to heterogeneous conjugates with undefined stoichiometry and sites, even with considerable batch-to-batch variability. In this study, we have developed a chemo-enzymatic strategy by equipping the C-terminus of anti-CD20 ofatumumab with a click handle using Sortase A, followed by ligation of the payload based on a strain-promoted azide-alkyne cycloaddition to produce homogeneous conjugates. The resulting antibody-drug conjugates fully retained their antigen binding capability and proved to be internalized and trafficked to the lysosome, which released the payload with a favorable efficacy in vitro and in vivo. Thus, this reported method is a versatile tool with maximum flexibility for development of antibody-drug conjugates and protein modification.

## 1. Introduction

Antibody-drug conjugates (ADCs) selectively deliver potent toxic payloads to antigen-positive tumor cells via specific antibody binding to reduce off-target toxicity and widen the therapeutic window in comparison to chemotherapeutics or combination therapy. Recent successes in the development of ADCs for targeted cancer therapy, such as Brentuximab vedotin (Adcetris^®^, SGN-35, Seattle Genetics, Inc., Bothell, WA, USA) and Trastuzumab emtansine (Kadcyla^®^, T-DM1, Genentech, Inc., San Francisco, CA, USA, have proven that ADCs can be potent weapons used in the battle against cancer [1,2,3,4].

All approved ADCs are produced by chemical conjugation that covalently attaches the toxic payload to the antibody through surface-exposed lysine or cysteine residues generated by reducing intrachain disulfide bonds of the antibody, which leads to heterogeneous mixtures in terms of position and stoichiometry of the payload coupled to the antibody (defined as “drug to antibody ratio”, DAR). Furthermore, different DAR of ADCs will have different efficacy, pharmacokinetic properties, stability and safety profiles [5], which result in a huge challenge in quality control. Consequently, diverse site-specific conjugation technologies have been further developed to produce homogeneous ADCs. One option is engineering free cysteines into antibodies which can react with maleimide-functionalized toxins, thereby yielding homogeneous ADCs with an improved therapeutic window [6]. Nonetheless, this approach requires a reduction-reoxidation procedure that can potentially lead to the formation of faulty intrachain or interchain disulfide bonds. Besides, the cysteine-maleimide linkages are instable in a circulatory system, because the maleimide-linker reaction can be exchanged by the active thiol group of cysteine in human serum, resulting in the premature release of toxins before internalization [7]. Thus, several site-specific conjugation approaches have been reported that utilize incorporated non-natural amino acids [8], or enzymatic modification, such as transglutaminase [9], formylglycine generating enzyme [10], and Sortase A (SrtA) [11].

**Figure 1:**
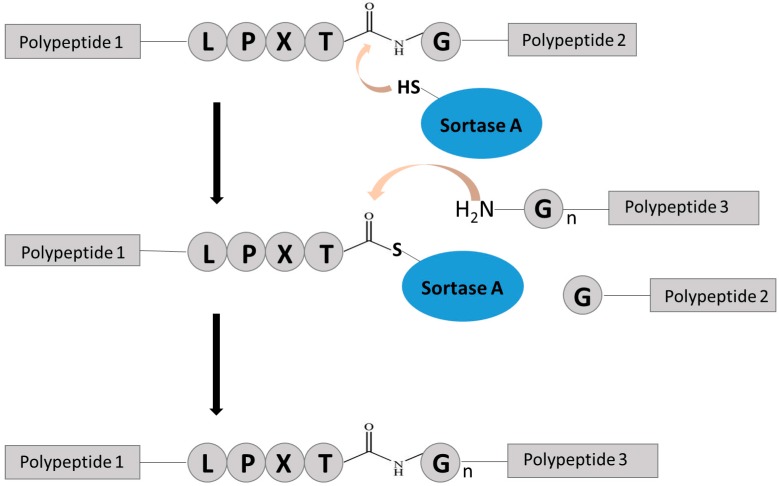
The reaction mechanism of Sortase A. Sortase A could recognize an LPXTG sequence (L, leucine; P, proline; T, threonine; G, glycine; X can be any amino acid), cleave the amide bond between the threonine (**T**) and the glycine (**G**) via an active-site Cys-184 and form a covalent thioester intermediate, then followed by the attack of oligoglycine substrates to produce covalently linked products.

SrtA could recognize substrates containing a highly conserved LPXTG (L, leucine; P, proline; T, threonine; G, glycine; X can be any amino acid)-sorting sequence motif, cleave the amide bond between the threonine (T) and the glycine (G), then, followed by the nucleophilic attack of oligoglycine substrates, produce covalently linked products (Figure 1). Owing to its high specificity and the wide range of oligoglycine substrates, SrtA has been extensively exploited for protein engineering and site-specific conjugation [12]. However, a huge excess of nucleophilic oligoglycine-modified toxins (100-fold molar excess) and enzyme are necessary to drive the equilibrium to form ADCs since the reaction is reversible [13]. Moreover, the toxins are usually expensive and cause the pollution with poisonous waste in the manufacturing process, losing its appeal in industrial large-scale production. In addition, the larger oligoglycine-functionalized toxins are less reactive than the small molecular weight isotype control substrate due to steric hindrance [14]. As an alternative, a chemo-enzymatic approach has shown potential value for the site-specific conjugation.

**Figure 2:**
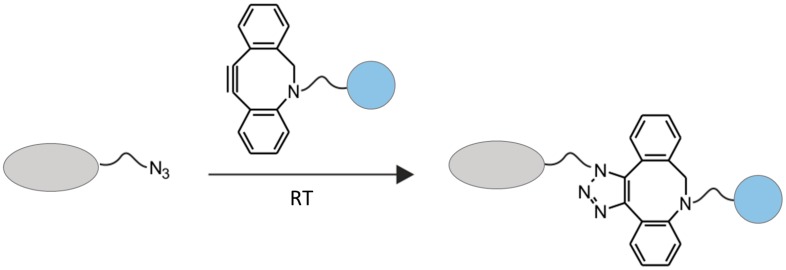
A reaction scheme of strain-promoted azide-alkyne cycloaddition. The azide was reacted with the dibenzylcyclooctyne (DBCO) in a Cu(I)-free [2 + 3] cycloaddition under mild conditions to produce 1,4-substituted triazoles.

**Figure 3:**
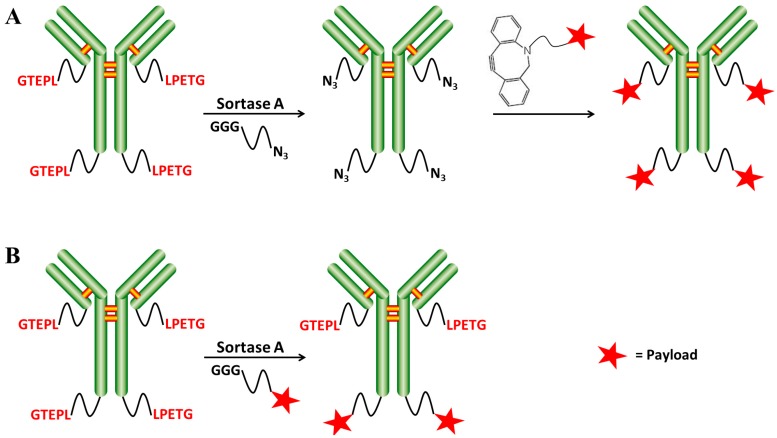
Schematic illustration of the two different strategies to generate homogeneous antibody-drug conjugates (ADCs). (**A**) For the chemo-enzymatic approach, a bifunctional oligoglycine-modified small molecule is enzymatically attached to the LPETG-tagged antibody in a first reaction. In a second step, the toxin, which is modified with a suitable functional group, is conjugated to the antibody via strain-promoted azide-alkyne cycloaddition (SPAAC); (**B**) The direct enzymatic approach includes direct conjugation of an oligoglycine-functionalized toxin to the LPETG-tagged antibody. LPXTG: L, leucine; P, proline; T, threonine; G, glycine; X can be any amino acid.

**Chart 1:**
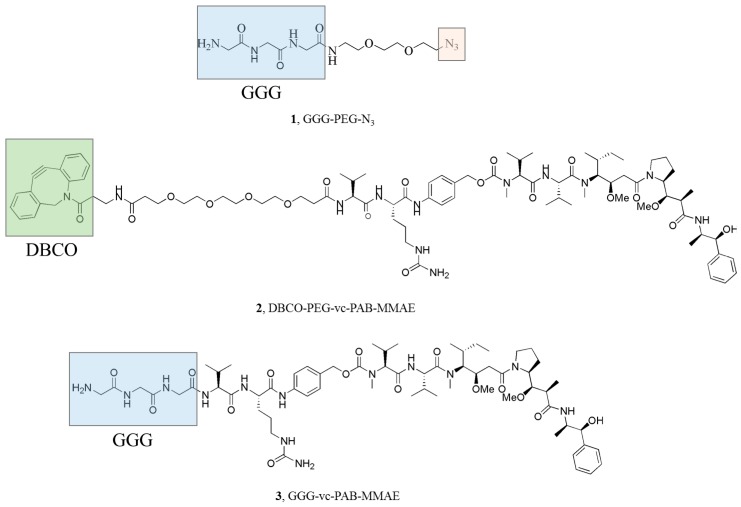
Chemical structures of the different substrates for the enzymatic and chemo-enzymatic approach. (**1**) GGG-PEG-N_3_ (GPN) is a bifunctional small molecule comprising an oligoglycine at the N-terminus and a bio-orthogonal azide functional group at the C-terminus through an oligoethylene glycol ligation; (**2**) DBCO-PEG-vc-PAB-MMAE was monomethyl auristatin E (MMAE) derivative containing a dibenzylcyclooctyne (DBCO), an oligoethylene glycol (PEG), a protease-sensitive valine-citrulline (vc) dipeptide and a self-immolative spacer, p-aminobenzylcarbamate (PAB); (**3**) GGG-vc-PAB-MMAE was modified by the addition of an oligoglycine to the vc-PAB-MMAE.

Click chemistry, especially the strain-promoted azide-alkyne cycloaddition (SPAAC), is an emerging tool for protein modification. The reaction of azide with strained cyclooctyne is highly specific, efficient, and proceeds under mild conditions (Figure 2). Herein, we presented a chemo-enzymatic approach using sortase-mediated ligation and Cu(I)-free click reaction (Figure 3A). In the first step, we introduced an easy-to-synthesize bifunctional oligoglycine-modified small molecule (GGG-PEG-N_3_, GPN, Chart 1, 1) comprising a bio-orthogonal azide functional group at the C-terminus through enzymatic catalysis to accomplish site-specific covalent attachment of functional molecules to the C-terminus of a fully humanized anti-CD20 antibody, ofatumumab (OFA), and subsequently reacted with a suitable monomethyl auristatin E (MMAE) derivative (DBCO-PEG-vc-PAB-MMAE, Chart 1, 2) via strain-promoted azide-alkyne cycloaddition to generate the desired ADC. Since the oligoglycine-modified small molecule (GGG-PEG-N_3_) and sortase enzyme are readily available in larger quantities from chemical synthesis and *E. coli* production respectively, it is conceivable that this design is suitable for scale-up production. Compared with the direct enzymatic attachment of toxic payloads to the antibody (Figure 3B), the chemo-enzymatic approach only required a 2.0 molar excess of toxin per azide group to yield homogeneous ADCs with a DAR of 3.3, which could be efficiently internalized by CD20-positive tumor cells even though the non-internalization of CD20 antigen was reported [15] and were highly efficacious in vitro and in vivo.

## 2. Results and Discussion

### 2.1. Preparation of Optimized OFA

**Figure 4:**
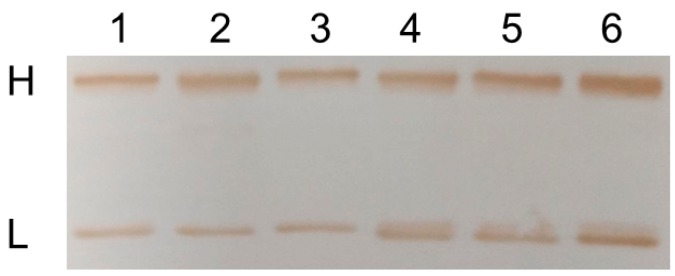
Conjugation efficiency of six ofatumumab (OFA) variants. Western blot analysis was performed for determination of monomethyl auristatin E (MMAE) levels of light and heavy chains. Lane 1, H/L-LPETG; Lane 2, H/L-G_4_SLPETG; Lane 3, H/L-(G_4_S)_2_LPETG; Lane 4, H/L-LPETGGH_6_; Lane 5, H/L-G_4_SLPETGGH_6_; Lane 6, H/L-(G_4_S)_2_LPETGGH_6_. H, heavy chain; L, light chain.

We previously have shown that the direct enzymatic conjugation using a 100-fold excess GGG-vc-PAB-MMAE (Chart 1, 3) yielded approximately 70% of modified heavy chain for OFA-vcMMAE (DAR = 1.4) [13]. However, the conjunction was very inefficient for the light chain, which may be not accessible to the light chain for the enzyme and the substrate due to steric hindrance. A flexible linker (G_4_S)_n_, often used to link the antibody formats [16], was inserted before the LPETG recognition motif (L, leucine; P, proline; E, glycine; T, threonine; G, glycine) at the C-terminus of light and heavy chains in order to improve the conjugation efficiency. Thus, six anti-CD20 OFA variants were engineered with a C-terminal (GGGGS)_n_LPETG(GHHHHHH)_m_ sequence (where *n* = 0, 1 or 2, and m = 0 or 1), and successfully expressed in HEK293F cells, which still remained the binding affinity to CD20 of cell surface. In order to seek the optimized antibody, six OFA variants were tested for the relative conjugation efficiency using chemo-enzymatic approach, and MMAE covalently coupled to the light and heavy chains was visualized with 3,3’-diaminobenzidine (DAB) color development after western blot of the conjugated antibody (Figure 4). In accordance with previous studies [17,18], the conjugation efficiency to the light chain and heavy chain was progressively improved by increasing short peptide spacer (G_4_S)_n_ length after the attachment of His-tag which will be lost in the enzymatic reaction. Therefore, we chose the (G_4_S)_2_LPETGGHHHHHH tagged OFA to conjugate toxins using chemo-enzymatic method to generate ADCs in this study.

### 2.2. Chemo-Enzymatic Approach to Produce ADC and Its Characterization

We developed a chemo-enzymatic approach by SrtA-mediated ligation and click chemistry to produce ADC (Table 1). In the first place, a small molecule substrate (GGG-PEG-N_3_, 2) suitable for the SrtA-catalyzed transpeptidation was used to transfer an additional azide functionality onto the antibody at a rate of 100:1 in order to promote enzymatic reaction, while the antibody formats (scFv or Fab) required less oligoglycine-modified substrate for their easy access [11]. In the second step, the toxin (DBCO-PEG-vc-PAB-MMAE, 3), which was equipped with an alkynyl group reactive to the azide, could then be coupled with the antibody by the strain-promoted azide-alkyne cycloaddition (SPAAC) under mild condition to yield the desired ADC. The SPAAC reaction was very fast and efficient with a minimal molar excess of toxin to yield homogeneous ADCs (2 molar equivalent per azide group), while direct SrtA-based attachment of MMAE derivative required a 100 molar excess of drug since toxins are expensive and may cause hazardous waste during the manufacturing procedure [13]. Moreover, the heterocyclic triazole linkage generated by SPAAC has been previously shown to be highly stable both in vitro and in vivo [19]. Recent studies that successfully employed enzyme-mediated ligation and click chemistry to prepare PEGylated capsules [20] and streptavidin-hydrogel [21], combined with our results, clearly demonstrate the broad utility of this technology. This chemo-enzymatic approach with flexibility and versatility could be readily adapted to a variety of antibodies and toxins, and correlational research is under investigation.

**Figure 5:**
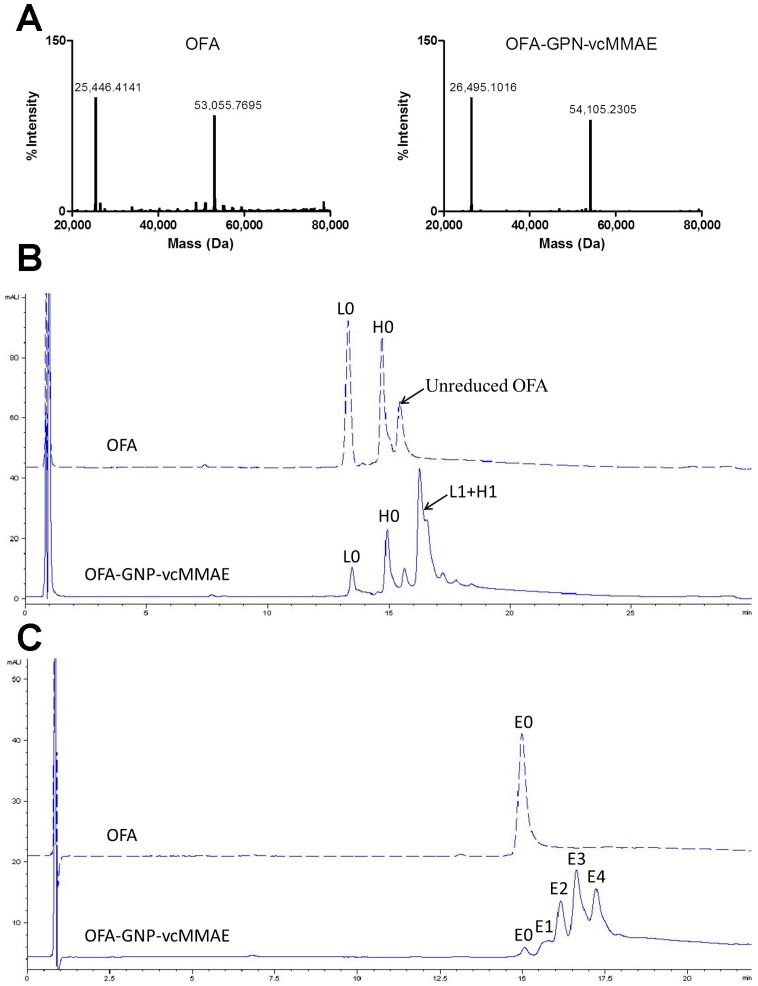
The characterization of chemo-enzymatic ADC. (**A**) Deconvoluted Q-TOF mass spectrum of ofatumumab (OFA) and OFA-GPN-vcMMAE. The mass peaks of light chain (25,446.4 Da) and heavy chain (53,005.8 Da) for OFA increase by 1049 Da after the chemo-enzymatic conjugation in accordance with the expected mass shift; (**B**) Reversed phase high performance liquid chromatography (RP-HPLC) evaluation of conjugation efficiency. Peak L0 and H0 indicate the light chain and heavy chain, and L1 and H1 represent the light chain and heavy chain conjugated with MMAE. Our chemo-enzymatic ADC was difficult to be completely reduced even in the presence of 100 mM dithiothreitol (DTT) due to stable structure, which is one possible explanation of the unreduced peak; (**C**) Hydrophobic interaction chromatography (HIC) analysis of the DAR under native condition. E0 (1, 2, 3 or 4) means no (one, two, three or four) MMAE molecule was conjugated to the intact antibody.

To confirm successful conjugation of MMAE via our chemo-enzymatic approach, the reduced molecular weights of the light chain and heavy chain for OFA and OFA-GPN-vcMMAE were analyzed by Q-TOF MS (Waters, Worcester, MA, USA) (Figure 5A). The mass differences of the light chain and heavy chain corresponded to the loss of the peptide cleaved during the enzymatic reaction (955 g/mol) plus the molecular weights of GGG-PEG-N_3_ (345 g/mol, 1) and DBCO-PEG-vc-PAB-MMAE (1657 g/mol, 2), approximately consistent with the expected mass shift of 1049 Da. As expected, we also confirmed the conjugation site on LPETG motif (Figure S1). Futhermore, the DAR was determined by reversed phase high performance liquid chromatography (RP-HPLC) (Figure 5B) and hydrophobic interaction chromatography (HIC) (Figure 5C). The chemo-enzymatic approach harvested a high yield of ADCs with a DAR of 3.3, about 75% conjugation efficiency at the heavy chain, and >90% conjugation efficiency at the light chain, achieving site-specific conjugation of drug to antibody, wherein both the modification sites and DARs could be more precisely controlled. Although the obtained ADC containing a small amount of lower DAR species, its homogeneity is higher than general ADCs constructed by traditional lysine or cysteine ligation [1,2,3,4].

### 2.3. ADC Binding to CD20-Positive Cells

**Figure 6:**
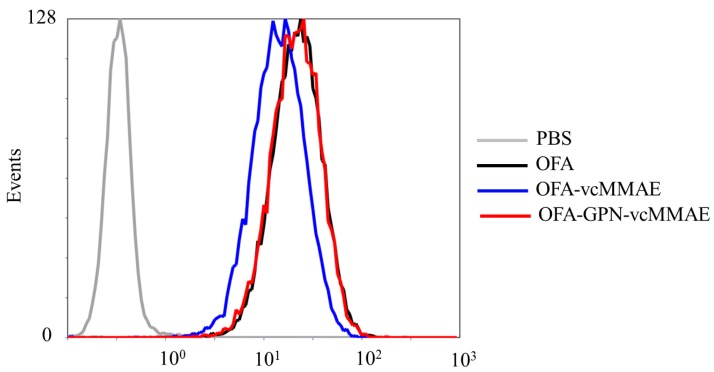
Determining the binding of antibody and ADCs to CD20-positive Daudi cells by flow cytometry. PBS: phosphate-buffered saline; OFA-vcMMAE: the direct enzymatic ADC.

To investigate the effects of the conjugation processes of toxins on targeted antigen-binding, the qualitative cell-binding experiments were performed with CD20-positive Daudi cells using flow cytometry. The ADCs were found to retain the antigen binding capability, which demonstrated that the modification of MMAE had minimal effect on the binding affinity to cell surface CD20 when compared with the unconjugated antibody (Figure 6). This result is not surprising because the attachment site of drug is distant from the antigen-binding site.

### 2.4. Internalization of ADC and Subcellular Localization

**Figure 7:**
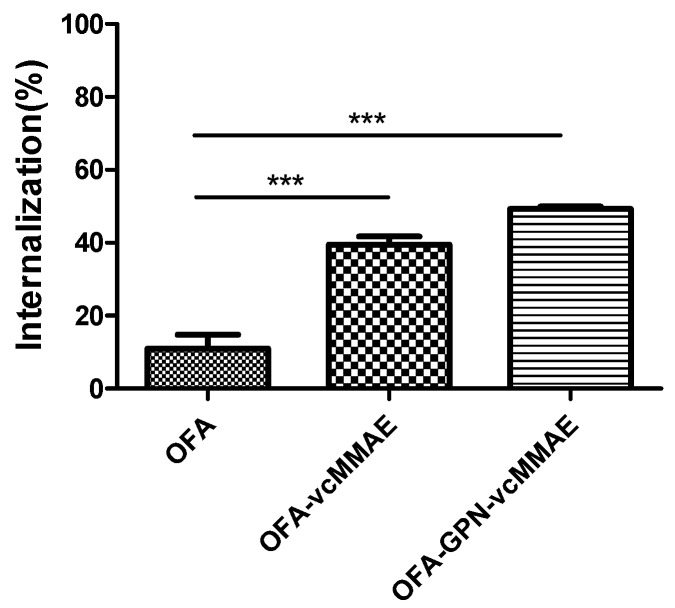
The percentages of internalization of OFA, OFA-vcMMAE and OFA-GPN-vcMMAE at 37 °C for 2 h. The *p*-value is indicated as follows: *** *p* <0.001, compared with unconjugated OFA group.

Next, we examined the internalization of OFA and the corresponding ADCs, which is a predominant factor in the function of ADC after antigen binding. Daudi cells were incubated for 30 min at 4 °C with serial dilutions of either the unconjugated OFA or the corresponding ADCs, stained with FITC-labeled secondary antibody, and examined by flow cytometry. Figure S2 showed that the relationship between the mean fluorescence intensity (MFI) and the concentration of antibody or ADC is linear within an applicable scope, suggesting that the internalization of the antibody and ADCs by the target cells could be evaluated by the mean fluorescent intensity of cell surface. Change in the surface levels of antibody or ADCs on Daudi cells was determined by flow cytometry after incubation at a concentration of 2 μg/mL for 2 h at 37 °C (Figure 7). In accordance with previous conclusions [22], cell surface loading with anti-CD20 antibody remained relatively constant after 2 h at 37 °C. Unconjugated anti-CD20 antibody was hard to be internalized into CD20-positive cells since CD20 is a noninternalizing antigen [23]. In contrast, fluorescent intensity of OFA-GPN-vcMMAE and OFA-vcMMAE declined significantly. Within 2 h, >40% and >30% of OFA-GPN-vcMMAE and OFA-vcMMAE, respectively, were internalized into the Daudi cells, which was significantly enhanced after conjugation with vcMMAE, suggesting that the conjugation of vcMMAE could promote the internalization of OFA in Daudi cells. Previous studies showed that internalization of type I anti-CD20 antibody was greatly augmented by their engagement with FcγRIIb on the cell surface via antibody bipolar bridging and the rate of internalization positively correlated with cell surface expression of FcγRIIb [24]. Hence, we speculated that vcMMAE could engage with FcγRIIb on Daudi cells, followed by the internalization of CD20-ADC-FcγRIIb complex. However, the biological mechanisms need to be further studied.

**Figure 8:**
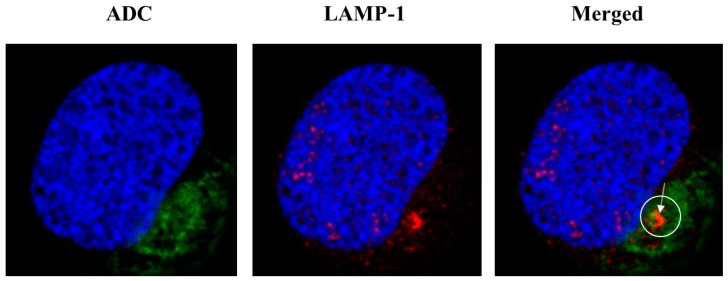
Trafficking and subcellular localization of OFA-GPN-vcMMAE. Daudi cells were treated with OFA-GPN-vcMMAE at 37 °C for 6 h. Cells were fixed, permeabilized, and cell-associated ADC and lysosome-associated membrane protein-1 (LAMP-1) were detected with respective fluorochrome-conjugated secondary antibodies. Nuclei were stained with DAPI (blue). Arrow indicated co-localization of ADC (green) with lysosomes (red).

Moreover, the subcellular trafficking and localization of OFA-GPN-vcMMAE in target cells were further determined using confocal microscope as shown in Figure 8. The ADC was localized inside the cells after 6 h cell incubation, verifying its internalization. Besides, the ADC co-localized with lysosomal-associated membrane protein-1 (LAMP-1), suggesting that OFA-GPN-vcMMAE appeared to be internalized and trafficked to the lysosome to release the payload. This result coincided with a previous study showing that the valine-citrulline (vc) dipeptide linkage with high stability in serum but only cleaved and efficiently released active drugs by lysosomal cathepsins [25].

### 2.5. Apoptosis and In Vitro Cytotoxicity

**Figure 9:**
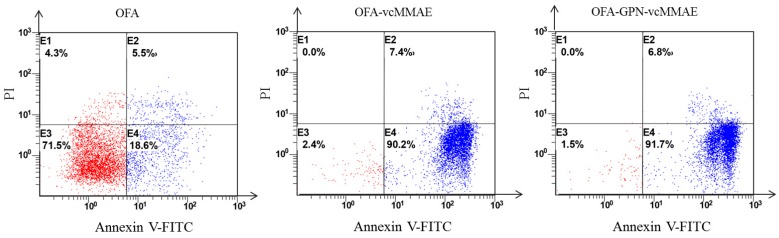
Effect of antibody and ADCs on induction of apoptosis in Ramos cells. Ramos cells were exposed to antibody, or ADCs at 5 μg/mL for 72 h. Induction of apoptosis was detected using Annexin V-FITC and PI-Staining by flow cytometry. Late apoptotic and necrotic cells: AnnexinV-FITC+/PI+ (upper-right). Early apoptotic cells: Annexin V-FITC+/PI− (lower right).

To study the cellular toxicity, the degree of apoptosis in Ramos cells was determined by Annexin V-FITC/PI staining after incubated with OFA or ADCs at a concentration of 5 μg/mL for 72 h, as shown in Figure 9. The percentages of OFA induced apoptotic cells (Annexin V+/PI−) and dead cells (Annexin V+/PI+) were merely 18.6% and 5.5%, respectively. In contrast, OFA-GPN-vcMMAE and OFA-vcMMAE induced >90% apoptotic cells and approximately 7% dead cells, suggesting the apoptosis-inducing ability of ADCs mainly depends on highly toxic MMAE, which has been reported to induce great growth arrest and apoptosis [26].

**Figure 10:**
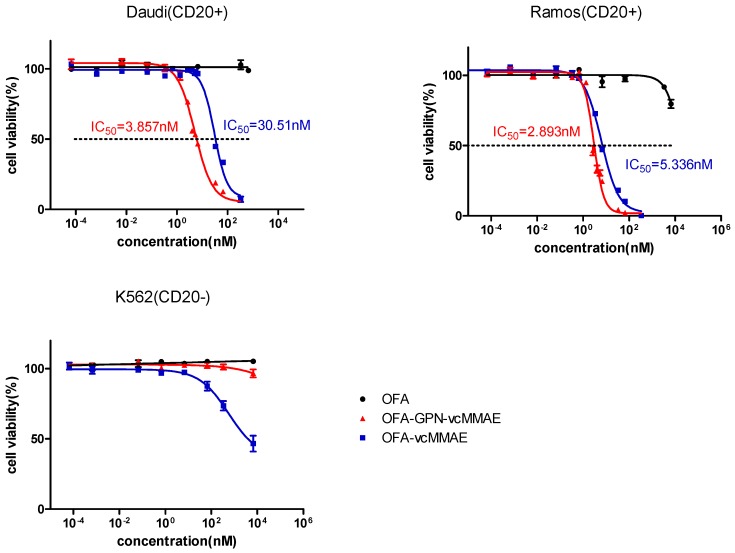
In vitro toxic effects of OFA-GPN-vcMMAE, OFA-vcMMAE and unconjugated OFA on CD20-positive (Daudi, Ramos) and negative (K562) cell lines. CD20+: CD20-positive; CD20-: CD20-negative.

Next, we further evaluated the cytotoxicity in vitro and selectivity of the ADC, two CD20-positive B-lineage lymphoma cell lines (Daudi, Ramos) and one CD20-negative cell line (K562) were included. The results showed that unconjugated OFA possessed very low toxicity on two CD20-positive cells (IC_50_ > 6.667 × 10^3^ nM) (Figure 10). In contrast, OFA-GPN-vcMMAE (DAR = 3.3) effectively killed Ramos and Daudi cells with an IC_50_ of 2.893 nM and 3.857 nM, respectively, while the direct enzymatic control OFA-vcMMAE (DAR = 1.4) was slightly less cytotoxic with corresponding IC_50_ of 5.336 nM and 30.51 nM. The potency of these ADCs correlated well with the DAR value. These IC_50_ values are comparable with published values about anti-CD20-based ADCs generated by reduced interchain disulfides [22,23]. Significantly, two anti-CD20-MMAE conjugates showed an approximately 1000-fold lower IC_50_ than unconjugated OFA against two CD20-positive cells, clearly demonstrating that the enhanced potency of the ADCs in comparison to the unmodified antibody. Moreover, both OFA-vcMMAE and OFA-GPN-vcMMAE showed far less potency on CD20-negative K562 cells compared to their effects on the CD20-positive cells, indicating the cytotoxicity is specific for CD20-positive cells.

### 2.6. In vivo Antitumor Activity

**Figure 11:**
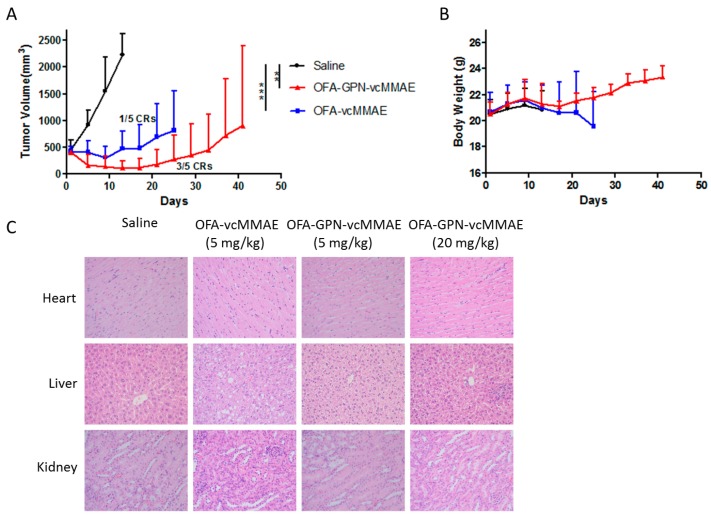
In vivo efficacy and toxicity of OFA-vcMMAE and OFA-GPN-vcMMAE in human tumor xenograft mouse models. (**A**) In vivo antitumor activities of OFA-vcMMAE and OFA-GPN-vcMMAE conjugates. The *p*-value is indicated as follows: ** *p* <0.01, *** *p* <0.001, compared with saline group. (**B**) Body weight monitoring of mice after drug administration; (**C**) Acute toxicity evaluation of mice treated with saline, OFA-vcMMAE (5 mg/kg) and OFA-GPN-vcMMAE (5 mg/kg or 20 mg/kg) via histological sections of the major organs (heart, liver and kidney) after H&E staining. Magnification was 40×.

The in vivo efficacy of chemo-enzymatic ADC was explored using a Ramos B-lymphoma xenograft model in nude mice. Saline solution was used as negative control, and direct enzymatic ADC was included as a comparator. When mean tumor size in each group reached approximately 450 mm^3^ (3–9 fold of the regular starting tumor volume of 50–150 mm^3^), therapy was initiated once every four days for four times (q4d × 4), and the tumor growth was monitored (Figure 11A). Tumors treated with the saline control grew rapidly and reached an average tumor volume of more than 2000 mm^3^. In contrast, OFA-GPN-vcMMAE and OFA-vcMMAE were able to significantly delay tumor growth (*** *p* < 0.001 and ** *p* < 0.01 compared with untreated control group). During the first week, OFA-GPN-vcMMAE and OFA-vcMMAE groups showed efficient tumor shrinkage. After one week, tumor sizes gradually increased for mice treated with OFA-vcMMAE but not for OFA-GPN-vcMMAE group, indicating OFA-vcMMAE showed lower potency in vivo compared with OFA-GPN-vcMMAE. Remarkably, the tumor disappeared completely in three of five mice in OFA-GPN-vcMMAE treated group, and no tumor recurrence was observed among them for the whole length of the study, leaving two slowly progressing.

At the same time, mice were weighed to evaluate the in vivo toxicity of ADC (Figure 11B). Slight weight loss was observed in ADC-treated groups compared with untreated group. However, the mice in OFA-GPN-vcMMAE group gradually returned to their normal weights after treatment, indicating the absence of severe or nonrecoverable toxicity. In addition, the significant reduction in body weights was continuing in OFA-vcMMAE group, indicating that the chemo-enzymatic ADC exerted improved therapeutic effect and decreased toxicity compared to direct enzymatic ADC.

For acute toxicity evaluation, mice were sacrificed 24 h after the last administration, and histological sections of the major organs (heart, liver and kidney) of the mice were examined after H&E staining. No obvious histomorphologic alterations were observed in any sections of organs except that hepatic steatosis was found in OFA-vcMMAE group (Figure 11C). To further evaluate the maximum tolerated dose (MTD) of our chemo-enzymatic ADC, we randomly selected a tumor-bearing nude mouse to treat intravenously high-dosage OFA-GPN-vcMMAE (20 mg/kg). No overt toxicity was found in the animal treated with 20 mg/kg OFA-GPN-vcMMAE (Figure 11C). Taken together, these suggested that a considerable therapeutic window may exist for OFA-GPN-vcMMAE. However, toxicology, pharmacokinetics and pharmacodynamics of ADC need to be further studied.

## 3. Conclusions

In this study, we have developed a convenient and simple two-step procedure consisting of SrtA-mediated conjugation followed by a strain-promoted click reaction for the production of the site-specific ADC, which can be efficiently internalized when targeting the noninternalizing CD20 antigen and exhibited potent antitumor activity. Our results demonstrated that the in vitro and in vivo efficacy of such chemo-enzymatic conjugates is superior to those prepared using a direct enzymatic coupling method based on the higher DAR, and revealed the two-step approach is versatile with the potential to be readily scaled up on account of cost reduction and less toxic waste. In addition, OFA-GPN-vcMMAE showed an excellent therapeutic effect on the B-lymphoma mice model for a better cure rate and significantly reduced side effects (Table 1). Furthermore, we expect this enzymatic-click strategy to find a diversity of applications from protein modifications to functionalized materials for use in drug delivery as well as diagnostics and imaging.

## 4. Materials and Methods

### 4.1. Reagents

GGG-PEG-N_3_ (1), DBCO-PEG-vc-PAB-MMAE (2), and GGG-vc-PAB-MMAE (3) were synthesized by Concortis (San Diego, CA, USA) and the purity was above 95% according to RP-HPLC analysis. Mouse polyclonal antibody against MMAE was produced and validated in the immunized mice from our laboratory. All reagents were purchased from Sigma-Aldrich (Saint Louis, MO, USA) unless otherwise noted.

### 4.2. Cell Lines and Cell Culture

Human CD20-positive (Daudi, Ramos) and CD20-negative (K562) cell lines were purchased from American Type Culture Collection (ATCC, San Francisco, CA, USA), and maintained in RPMI-1640 medium (GIBCO, Grand Island, NY, USA) supplemented with 10% fetal bovine serum (FBS, GIBCO) at 37 °C in 5% CO_2_ atmosphere.

### 4.3. Expression and Purification of Recombinant SrtA

A DNA sequence encoding residues 60–206 of the *S. aureus* SrtA (∆N59) was synthesized, inserted into pET28a (+) vector, and expressed in *E. coli* Rosetta (DE3) as a fusion protein with an C-terminal His-tag, followed by the purification as described previously [13]. The amino acid sequence of SrtA (∆N59) as followed:

QAKPQIPKDKSKVAGYIEIPDADIKEPVYPGPATPEQLNRGVSFAEENESLDDQNISIAGHTFIDRPNYQFTNLKAAKKGSMVYFKVGNETRKYKMTSIRDVKPTDVGVLDEQKGKDKQLTLITCDDYNEKTGVWEKRKIFVATEVK.

### 4.4. Production and Purification of Tagged Antibodies

The full-length human light and heavy chain sequences of OFA [13] were tagged with a SrtA recognition sequence (LPETG) at the C-terminus followed by a His-tag, and then inserted into the GC-rich eukaryotic expression vector pMH3 plasmids (AmProtein, Hangzhou, China) for expressing full-length human light and heavy chains. Moreover, the signal peptide sequence (MDWTWRILFLVAAATGAHS) was genetically fused to the N-terminus of light and heavy chain, and the (GGGGS)_n_ sequence was inserted before the LPETG sequence using standard molecular biology techniques. Thus, the sequence variants appended to the C-terminus of the light and heavy chain were (GGGGS)_n_LPETG(GHHHHHH)_m_ (*n* = 0, 1 or 2, and m = 0 or 1). Finally, sequences were confirmed by DNA sequencing (Sangon Biotech, Shanghai, China). Primers used above are detailed in Table S1 in the Supplementary Materials.

For the expression of the tagged antibodies, relevant expression plasmids were transiently transfected into human embryonic kidney cells (HEK293F) in suspension culture with polyethylenimine as described previously [27]. The recombinant antibodies were purified by chromatography using a Ni-NTA or Protein A purification column (GE Healthcare, Pittsburgh, PA, USA). The buffer of eluted antibodies was exchanged to 50 mM Tris-HCl (pH 7.5), 150 mM NaCl by ultrafiltration (Amicon Ultra-30k, Millipore, Billerica, MA, USA), and stored at −80 °C until further use.

### 4.5. SrtA-Mediated Conjugation

OFA with C-terminal LPETG sequence (2 μM) was conjugated to GGG-PEG-N_3_ (200 mΜ, 1) in the presence of SrtA (50 μM) in reaction buffer (50 mM Tris, 150 mM NaCl, 5 mM CaCl_2_, pH 7.5) for 12 h at 37 °C. The His-tag on the OFA was removed during this reaction. The azide-functionalized antibodies (OFA-GPN) were purified by protein A chromatography. The buffer of eluted antibodies was exchanged to 50 mM Tris-HCl (pH 7.5), 150 mM NaCl by ultrafiltration, and sterile filtered and stored at −80 °C.

### 4.6. Click Chemistry Reaction

For the strain-promoted azide-alkyne cycloaddition (SPAAC), OFA-GPN was incubated with DBCO-PEG-vc-PAB-MMAE (2 molar equivalent per azide group, 2), adjusted to pH 7.4 in PBS at RT overnight. After the incubation, the samples were washed repeatedly with PBS by ultrafiltration to remove excess of DBCO-PEG-vc-PAB-MMAE (2), and sterile filtered and stored at −80 °C.

### 4.7. Western Blot

Equivalent proteins were separated with 10% sodium dodecyl sulfate-polyacrylamide gel electrophoresis (SDS-PAGE) and electroblotted onto polyvinylidene fluoride (PVDF) membranes, followed by the incubation with blocking solution containing phosphate-buffered solution (PBS) (pH 7.4) with 5% skimmed milk and 0.1% Tween-20 for 1 h at 37 °C, and then with an anti-MMAE primary antibody (1:1000 dilution) for 2 h at 37 °C. Blots were then washed three times with 0.1% Tween-20/PBS (TPBS), incubated with a 1:1000 dilution of horse radish peroxidase (HRP)-labeled secondary antibody (Beyotime, Shanghai, China) for 2 h at 37 °C and washed three times with TPBS again. Protein bands were visualized with DAB Horseradish Peroxidase Color Development Kit (Beyotime, Shanghai, China).

### 4.8. Liquid Chromatography-Mass Spectrometry (LC-MS)

Analysis was done on a Waters Xevo-G2S Q-TOF mass spectrometer coupled to a Waters ACQUITY UPLC. Samples were diluted to a final concentration of 2 mg/mL, and then the disulfide bonds were reduced in the presence of 12 mM DTT at 37 °C for 40 min. Desalting and chromatographic separation were performed using a 12 min linear gradient between 10% B and 90% B. (mobile phase A: 0.1% formic acid in water; mobile phase B: acetonitrile with 0.1% formic acid). Mass spectrometry parameters were as followed: capillary (kV), 2.5; sampling cone (V), 60; source temperature (°C), 120; desolvation temperature (°C), 500; desolvation gas flow (L/h), 800; mass range (*m*/*z*), 2000–8000.

### 4.9. Reversed Phase High Performance Liquid Chromatography (RP-HPLC)

RP-HPLC was performed using a Varian PLRP-S column (100 Å, 8 μm, 150 × 25 mm). As elution buffer A, 0.1% trifluoroacetic acid in water was used. Elution buffer B was composed of acetonitrile, exclusively. Elution conditions were as followed: 0–3 min 25% buffer B; 3–25 min 25%~50% buffer B; 25–27 min 50%~95% buffer B; 27–28 min 95%~25% buffer B; 28–30 min 25% buffer B at 0.6 mL/min. Samples were previously reduced by incubation with DTT at 37 °C for 30 min before performing RP-HPLC.

### 4.10. Hydrophobic Interaction Chromatography (HIC)

HIC was performed on a TOSOH Butyl-NPR column (2.5 μm, 4.6 mm × 3.5 cm) at 0.8 mL/min with a 15 min linear gradient between elution buffer A-1.5 M (NH_4_)_2_SO_4_, 25 mM Na_3_PO_4_, pH 7.0 and elution buffer B-75% 25 mM Na_3_PO_4_, pH = 7.0, 25% isopropanol (IPA).

### 4.11. Cell Binding

Binding of antibody or ADCs to cell-surface CD20 was assessed by flow cytometry (FC500MCL, Beckman Coulter, Brea, CA, USA) on CD20-positive Daudi cells. 1 × 10^6^ cells were incubated with 5 μg/mL antibody or ADCs in staining medium containing PBS (pH 7.4) with 1% (*w*/*v*) bovine serum albumin (BSA) on ice for 30 min, and then washed twice with ice-cold PBS to remove unbound antibody or ADCs. Cells were stained with FITC-labeled secondary antibody (Beyotime, Shanghai, China) at a dilution of 1:250 in ice-cold staining medium, incubated 30 min on ice, and washed as above. Labeled cells were examined by flow cytometry gated to exclude nonviable cells. Data was analyzed using CXP Analysis 2.2 (Beckman Coulter, Brea, CA, USA) and the background-corrected mean fluorescence intensity was determined.

### 4.12. Cellular Internalization

Briefly, Daudi cells were incubated with serial dilutions of antibody or ADCs on ice for 30 min and then washed twice. Next, cells were incubated with fluorescein isothiocyanate (FITC)-labeled secondary antibody for 30 min on ice, followed by the analysis of mean fluorescence intensity (MFI) after wash. The MFI-concentration curve was produced, and the linear range was selected as the standard curve, indicating that the percentage of internalization could be calculated by MFI within this range. Simultaneously, cells in triplicate were incubated with 2 μg/mL antibody or ADCs on ice for 30 min. After washing, cells were incubated at 37 °C for 2 h to drive internalization, and then cell supernatants were collected after centrifugation. Antibody or ADCs may be shed from the cell surface to the media, and Daudi cells were incubated with the centrifuged supernatants containing antibody or ADCs on ice for 30 min. Then cells surface-bound antibody or ADCs were stained with FITC-labeled secondary antibody and analyzed by flow cytometry after wash. The concentration was calculated according to the linear MFI-concentration standard curve after subtracting the background value of MFI derived from the untreated control. The volume of incubation above was fixed to 200 μL. The percentage of internalization of antibody or ADCs was calculated based on the following formula: Internalization (%) = (4 °C total surface-bound amount −37 °C total surface-bound amount −37 °C dissociative amount from the surface to media)/4 °C total surface-bound amount ×100%.

### 4.13. Microscopy for ADC Trafficking

Daudi cells were plated on 12-well plates (Corning, Corning City, NY, USA) at a density of 5 × 10^5^ cells/mL and treated with 5 μg/mL ADC at 37 °C with 5% CO_2_. After a 6-h incubation, the cells were gently washed twice with PBS (pH 7.4) to remove unbound ADC, and then seeded on slides by centrifugation. Cells were fixed with 4% paraformaldehyde solution for 15 min. After PBS washing, cells were permeabilized for 10 min with 0.1% Triton X-100, 0.2% bovine serum albumin (BSA)-PBS, followed by a blocking step with 2% BSA-PBS for 30 min. After the blocking, cells were stained by rabbit anti-lysosome-associated membrane protein-1 (LAMP-1) antibody (Abcam, Cambridge, UK) at a dilution of 1:500 in 1% BSA-PBS (pH 7.4) for 45 min. After gentle washing, cells were stained by FITC-labeled goat anti-human IgG (H + L) polyclonal antibody (Beyotime, Shanghai, China) and Cy5-labeled goat anti-rabbit IgG (H + L) polyclonal antibody (Beyotime, Shanghai, China) for 45 min, and then gently washed. Nuclei were further stained with DAPI for 3 min, and excessive dye was washed away. After staining, cells were covered with coverslip. Fluorescence images of the optical section were acquired with a Zeiss LSM 800 confocal laser scanning microscopy (Zeiss, Jena, Germany) and processed with ZEN 2 (blue edition) image analysis software (Zeiss, Jena, Germany).

### 4.14. Assessment of Apoptosis

Apoptosis and cell death were assessed with the Annexin V-FITC Apoptosis Detection Kit (Dojindo, Kumamoto, Japan). Ramos cells were seeded at a density of 5 × 10^4^ cells/mL in a 6-well plate (Corning, Corning City, NY, USA), and then exposure to 5 μg/mL antibody or ADCs for 72 h. At 72 h post-exposure, cells were stained with Annexin V-FITC and PI for apoptosis analysis. The percentages of apoptotic cells (AnnexinV+/PI−) and dead cells (AnnexinV+/PI+) were determined by flow cytometric analysis of each population.

### 4.15. In Vitro Cytotoxicity

In vitro potency was assessed using Cell Counting Kit-8 (CCK-8, Dojindo, Kumamoto, Japan) to determine cell viability on two CD20-positive tumor cell lines (Daudi, Ramos) and one CD20-negative tumor cell line (K562). Cells were cultured into log-phase growth, and then plated on 96-well plates (Corning, Corning City, NY, USA) in 100 μL growth medium (RPMI-1640 with 10% FBS) at a density of 10,000 cells/well and incubated at 37 °C in a humidified incubator in a 5% CO_2_ atmosphere. After 24 h, a serial dilution of antibody or ADCs in growth medium were added, resulting in final concentrations ranging from 10^−3^ ng/mL to 10^6^ ng/mL. The PBS (pH 7.4) was used as negative control. After exposure to antibody or ADCs for 96 h, cells were incubated with 10% CCK-8 agent for 4 h, and then the absorbance at 450 nm was measured by the BioRad Model 680 Microplate Reader. GraphPad Prism software was used for data analysis, including IC_50_ calculations.

### 4.16. In Vivo Antitumor Activity

To establish a localized xenograft model of human B-lymphoma, 8 × 10^6^ Ramos cells were implanted subcutaneously into the right flank of female Balb/c nude mice (Slaccas, Shanghai, China). When the tumors reached an average volume of 450 mm^3^, the animals were divided into six per group treated intravenously with saline, OFA-vcMMAE (5 mg/kg) and OFA-GPN-vcMMAE (5 mg/kg) respectively once every four days for four times (q4d × 4). The body weights of mice in each group were continuously monitored until the end of the experiment, and tumor volumes (mm^3^) were calculated according to the formula: V = (L × W^2^)/2, where L and W refer to the tumor length and width (mm), respectively.

Possible heart, liver or kidney toxicity was examined by morphological examination of relevant tissue cells after H&E staining. Briefly, tissues for H&E staining were obtained from experimental groups randomly (one mouse per group), 24 h after the last administration of ADCs. The heart, liver and kidney tissues obtained were fixed in 10% neutral buffered formalin, paraffin-embedded and stained by H&E. The sections were examined using light microscopes at 40× magnification. The histology was evaluated by a trained pathologist. To further determine the maximum tolerated dose (MTD), we randomly selected a tumor-bearing nude mouse to treat intravenously 20 mg/kg OFA-GPN-vcMMAE (q4d × 4) and were examined by histopathology as described above. All procedures related to animal handling, care, and the treatment in this study were performed in accordance with the National Institute of Health Guide for the Care and Use of Laboratory Animals. The protocols were approved by the Committee on the Ethics of Animal Experiments of the Zhejiang University, China (SCXK 2007-0029).

### 4.17. Statistical Analysis

Differences between the experimental groups were tested for significances on the basis of one-way analysis of variance (ANOVA), followed by Dunnett’s multiple comparison test using GraphPad Prism software (GraphPad Software, La Jolla, CA, USA). A probability value of less than 0.001, 0.01 and 0.05 (* *p* < 0.05, ** *p* < 0.01 and *** *p* < 0.001) was accepted as a significant difference.

## Figures and Tables

**Table 1:** The comparison of the different synthesis methods.

Characterizations	Enzymatic ADC	Chemo-Enzymatic ADC
Derivative payloads	100-fold molar excess	2 molar equivalent per azide group
DAR	1.4	3.3
Internalization (%)	39.44	49.31
IC_50_ on Ramos (nM)	5.336	2.893
IC_50_ on Daudi (nM)	30.51	3.857
Effect in vivo	*** *p* < 0.001 ^1^	** *p* < 0.01 ^1^
Safety in vitro and in vivo	lower	higher

^1^ *** *p* < 0.001 and ** *p* < 0.01 compared with untreated control group. ADC: antibody-drug conjugate; DAR: drug to antibody ratio.

## Supplementary Materials

Supplementary materials can be found at www.mdpi.com/1422-0067/18/11/2284/s1.

## Abbreviations

ADC	Antibody-drug conjugate
OFA	Ofatumumab
SrtA	Sortase A
SPAAC	Strain-promoted azide-alkyne cycloaddition
DAR	Drug to antibody ratio
MMAE	Monomethyl auristatin E
FBS	Fetal bovine serum
PBS	Phosphate buffered saline
BSA	Bovine serum albumin
MFI	Mean fluorescence intensity
LAMP-1	Lysosomal-associated membrane protein-1
