# Supplementary material for: A Versatile Chemo-Enzymatic Conjugation Approach Yields Homogeneous and Highly Potent Antibody-Drug Conjugates
Source: Int J Mol Sci. 2017 Oct 31;18(11):2284. doi: 10.3390/ijms18112284 (PMC5713254; doi:10.3390/ijms18112284)
Supplement: Supplementary file 1 [file ijms-18-02284-s001.docx]

Supplementary Materials: A Versatile Chemo-Enzymatic Conjugation Approach Yields Homogeneous and Highly Potent Antibody-Drug Conjugates

Ying Xu, Shijie Jin, Wenbin Zhao, Wenhui Liu, Ding Ding, Jie Zhou and Shuqing Chen

Supplementary Methods

Liquid Chromatography-Mass Spectrometry (LC-MS)

Analysis was done on a Waters Xevo-G2S Q-TOF mass spectrometer coupled to a Waters ACQUITY UPLC. Samples were diluted to a final concentration of 2 mg/mL by 6 M guanidine hydrochloride, 100 mM Tris-HCl (pH 8.0). The disulfide bonds were reduced in the presence of 12 mM DTT at 37 °C for 2.5 h, followed by the addition of iodacetamide to a final concentration of 60 mM, and incubated in dark for 40 min at RT. After the buffer was exchanged to 100 mM sodium phosphate (pH 7.4) via ultrafiltration, samples were digested by trypsin at 37 °C for 24 h, and then the digestion was stopped by adding formic acid to a final concentration of 1% (v/v). Desalting and chromatographic separation of digest peptides was performed using a 12 min linear gradient between 5% B and 95% B. (mobile phase A: 0.1% formic acid in water; mobile phase B: acetonitrile with 0.1% formic acid). Mass spectrometry parameters were as followed: capillary (kV), 2.0; sampling cone (V), 150; source temperature (°C), 90; desolvation temperature (°C), 400; desolvation gas flow (L/h), 700; mass range (m/z), 2000–8000.

Supplementary Figures

The corresponding full gene for recombinant antibodies was amplified by PCR to construct the protein with a C-terminal (GGGGS)_n_LPETG(GHHHHHH)_m_ sequences. Following primers (Sangon Biotech, Shanghai, China) were used:

**Table S1.** Primers for LPETG-tagged antibody.

| **No** | **Primer Name** | **Sequence (5’-3’)** |
| --- | --- | --- |
| 1 | H-up1 | TGGTGGCCGCCGCCACCGGCGCCCACTCCGAAGTGCAGCTGGT |
| 2 | L-up1 | TGGTGGCCGCCGCCACCGGCGCCCACTCCGAAATTGTGTTGAC |
| 3 | H/L-up2 | GCGAATTCACCACCATGGACTGGACCTGGCGGATCCTGTTCCTGGTGGCCGCCGCCA |
| 4 | H-LPETG-dn | ATTTGCGGCCGCTTAACCAGTCTCCGGCAGTTTACCCGGAGACA |
| 5 | H-G_4_SLPETG-dn | ATTTGCGGCCGCTTAACCAGTCTCCGGCAGAGAGCCTCCACCGCCTTTACCCGGAGACA |
| 6 | L-LPETG-dn | ATTTGCGGCCGCTTA ACCAGTCTCCGGCAGACACTCTCCCCTGT |
| 7 | L-G_4_SLPETG-dn | ATTTGCGGCCGCTTA ACCAGTCTCCGGCAGAGAGCCTCCACCGCCACACTCTCCCCTGT |
| 8 | H-(G_4_S)_2_LPETG-dn1 | ACCAGTCTCCGGCAGAGAGCCTCCACCGCCAGAGCCTCCACCGCCTTTACCCGGAGACA |
| 9 | L-(G_4_S)_2_LPETG-dn1 | ACCAGTCTCCGGCAGAGAGCCTCCACCGCCAGAGCCTCCACCGCCACACTCTCCCCTGT |
| 10 | H/L-(G_4_S)_2_LPETG-dn2 | ATTTGCGGCCGCTTAACCAGTCTCCGGCAGAGAGCCTCCACCGCC |
| 11 | H/L-LPETGGH_6_-dn | ATTTGCGGCCGCTTAGTGGTGGTGGTGGTGGTGACCACCAGTCTCCGGCAG |

Recognition sites for restriction enzymes are underlined (GAATTC for *EcoR I* , GCGGCCGC for *Not I*).


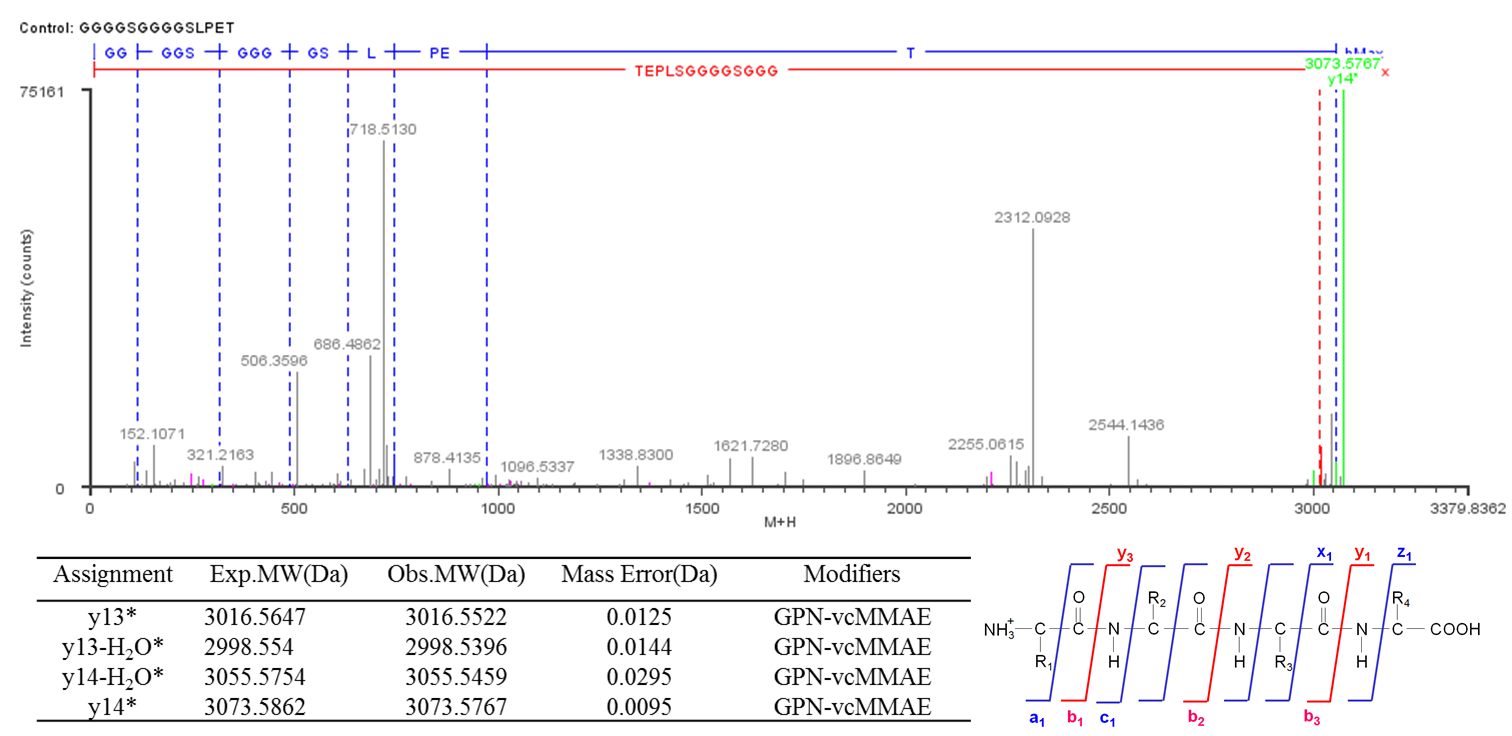


**Figure S1.** LC-MS analysis of the conjugation site on LPETG motif. The amino-acid sequence assignment of potential LPET-GPN-vcMMAE fragment. The ladder-like a-ion (-C-C) and b-ion (-C-N) series of peptide-fragment ions plus large modified moiety (y13 or y14, C-N-) facilitated the assignment of the amino-acid sequence (LPET) and the modified sites.


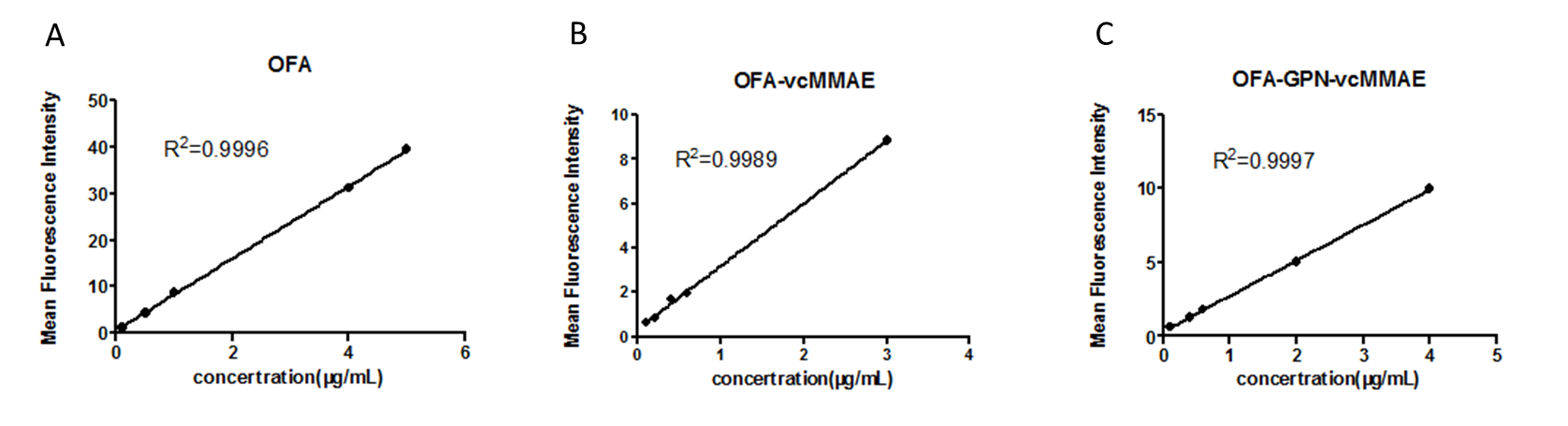


**Figure S2.** The linear relationship within an applicable scope between the mean fluorescence intensity and the concentration of (**A**) OFA, (**B**) OFA-vcMMAE and (**C**) OFA-GPN-vcMMAE.
